# Antimicrobial Activity of Copper Alone and in Combination with Lactic Acid against *Escherichia coli* O157:H7 in Laboratory Medium and on the Surface of Lettuce and Tomatoes

**DOI:** 10.4061/2011/650968

**Published:** 2011-10-23

**Authors:** Rabin Gyawali, Salam A. Ibrahim, Salma H. Abu Hasfa, Shahnaz Q. Smqadri, Yosef Haik

**Affiliations:** ^1^Food Microbiology and Biotechnology Laboratory, North Carolina Agricultural and Technical State University, 173 Carver Hall, Greensboro, NC 27411, USA; ^2^LFRD Department, ALCDR Institute, City of Scientific Research and Technology Applications, P.O. Box 21934, Alexandria, Egypt; ^3^Center of Research Excellence in Nanobioscience, The University of North Carolina Greensboro, Greensboro, NC 27412, USA

## Abstract

The objective of this study was to evaluate the effect of copper alone and in combination with lactic acid against *E. coli* O157:H7 in laboratory medium and on the surface of lettuce and tomatoes. Four strains of *E. coli* O157:H7 were individually inoculated into BHI broth containing different concentrations of copper (5, 10, 20, and 40 ppm, w/v), lactic acid (0.1 and 0.2%, v/v), and their combinations. After incubation, aliquots of 1 mL from each sample were withdrawn and plated on BHI agar to determine the bacterial population. Significant growth inhibition (*P* < 0.05) was observed with a combination treatment of copper (40 ppm) and lactic acid (0.2%). The population of *E. coli* O157:H7 was reduced by 3.93 and 3.39 log on the surface of lettuce and tomato samples, respectively, when treated with the same combination. This indicates that combination of copper and lactic acid could be used as an effective solution to inhibit *E. coli* O157:H7 on fresh produce.

## 1. Introduction

Fresh produce is a vital part of the human diet, but concerns about its safety are on the rise. Foodborne illness outbreaks linked to the presence of *E. coli* O157:H7 in fresh produce have increased over the past few years [1, 2]. Contaminated fresh produce thus represents the second leading cause of foodborne illnesses in the United States [3]. Different chemical agents (chlorine, hydrogen peroxide, organic acids, ozone, etc.) have been used to decontaminate fresh produce surfaces, but these treatments cannot completely inhibit bacterial pathogens [4, 5]. Among these treatments, a solution of chlorine is the most common and widely used chemical sanitizer in the fresh produce industry. However, treatment of produce with chlorinated water has a limited bactericidal effect and gives rise to other concerns due to its potential to form carcinogenic by-products [6–8]. Hence, there is a need for safer and more effective antimicrobial treatments for fruits and vegetables. 

Consumers are now paying closer attention to the use of artificial chemical preservatives to control foodborne pathogens. The food industry is also looking to use better ingredients as antimicrobial compounds to ensure the safety of food products. Two of the treatments considered in this study are copper and lactic acid. Copper is considered safe to humans as demonstrated inter alia by the use of copper intrauterine devices [9]. It is one of the relatively small groups of metallic elements essential to human health. Copper is required in trace amounts for the growth and functioning of microorganisms; however, higher concentrations can produce a toxic effect [10]. Copper ions have been reported to have antimicrobial activity against a wide range of microorganisms, including *Salmonella enterica*, *Campylobacter jejuni*,* E. coli*,* Listeria monocytogenes*, and* Staphylococcus aureus *[11–13]. Metallic copper surfaces have potential application as inhibitory agents in the various stages of food processing operations [11]. Copper has also been shown to inhibit germination, vegetative growth, and sporulation of *Clostridium tyrobutyricum* and has reduced the risk of late blowing spoilage in cheese indicating its potential as a food additive [14]. Lactic acid is generally recognized as safe and is widely used as a biopreservative in naturally fermented products [15]. Lactic acid solutions are one of the most widely applied organic acid treatments used to decontaminate foods. For example, lactic acid has been used successfully to decontaminate meat [16–19]. Lactic acid is therefore considered to have great potential to inactivate microorganisms on the surface of fruits and vegetables. 

Although information concerning the use of copper and lactic acid to decontaminate bacterial populations on fresh produce is not available, several studies have shown a synergistic inhibition effect on microbial growth. Copper sulfate (50 ppm) and lactic acid (150 mM) added to liquid pig feed were found to cause a 10-fold decrease in the D-value of *Salmonella typhimurium* DT104:30 [20]. In another study, a complete elimination of *Cronobacter *was obtained with a combination of copper (50 *μ*g mL^−1^) and lactic acid (0.2%) [21]. In a study using apple cider, a 5-log reduction was achieved for *E. coli* O157:H7 and *Listeria monocytogenes* with the combination of copper and sodium hypochlorite following sonication [13]. Similarly, Ibrahim et al. [12] reported a significant growth inhibition of *E. coli *O157:H7 and *Salmonella* when both copper (50 ppm) and lactic acid (0.2%) were added to laboratory medium and carrot juice. 

The mode of action underlying copper and lactic acid antimicrobial effects is not well understood. Studies in the past have proposed several mechanisms to explain the antibacterial activity of copper and lactic acid [22, 23] with regard to morphological changes in bacterial cells. Several techniques have been used to determine the effect of treatments on morphology of the cells. Scanning electron microscopy (SEM) is used for studying microbial colonization and attachments to food or food contact surfaces [24]. SEM is a common technique used to investigate the microorganism cell damage produced by antimicrobial compounds. The outer membrane permeability of Gram-negative bacteria is increased when the cells are exposed to an acidic substance such as lactic acid [25]. Based on this information, we hypothesized that this effect on permeability could enhance the antibacterial efficacy of copper in treating Gram-negative bacteria. The objectives of this study were thus to (i) establish a concentration of copper in combination with lactic acid that effectively inhibits the growth of *E. coli* O157:H7 in laboratory medium and (ii) determine if such concentration could be used to decontaminate *E. coli* O157:H7 populations on the surface of lettuce and tomatoes. 

## 2. Material and Methods

### 2.1. Bacterial Strains

The four strains of *E. coli* O157:H7 (H1730, 43895+, 43895−, and 86.24) used in this study were obtained from the Department of Food Science and Technology at Virginia Polytechnic Institute and State University (Blacksburg, Va, USA). These strains were maintained on tryptic soy agar slants at 4°C. Strains were then transferred to fresh brain heart infusion broth (BHI; Becton Dicksinson, Sparks, Md, USA) and incubated at 37°C for 12 h. Bacterial strains were then streaked onto a BHI agar plate and incubated at 37°C for 24 h. Single colonies of each strain were then transferred into BHI broth and incubated at 37°C for 12 h before use. 

### 2.2. Inoculum Preparation

Each bacterial strain was harvested by centrifugation (8,000 g for 10 min, 4°C). Cells in pellets were resuspended in 9 mL of sterile peptone solution (0.1%), serially diluted, and 100 *μ*L was inoculated into each treatment sample to achieve an initial inoculum level of approximately 3 log CFU/mL. To determine the effect of copper and lactic acid on the bacterial population on produce surfaces, separate cultures of each individual strain grown in 10 mL of BHI were mixed together (40 mL) to produce a four strain mixture of *E. coli* O157:H7. The cells of this mixture were harvested by centrifugation at 8,000 g for 15 min at 4°C. The supernatant was decanted, and the cell pellet was resuspended in 400 mL of sterile peptone solution (0.1%, w/v) to give a cell number of approximately 9 log CFU/mL. Bacterial populations in the inoculum were determined by surface plating duplicate samples on BHI after serial dilutions in peptone solution (0.1%, w/v). The plates were incubated for 24 h at 37°C, and then colonies were counted. 

### 2.3. Media Preparation

Seven ml batches of fresh BHI broth containing copper (stock solution of 3.95 g CuSO_4_·5H_2_O was prepared by dissolving in 1000 mL of sterilized distilled water to give 1000 ppm of copper) at concentrations of 5, 10, 20, and 40 ppm (w/v), lactic acid at 0.1 and 0.2%, v/v (Thermo Fisher Scientific, Fair Lawn, NJ, USA), and their combination were prepared. An additional 7 mL of BHI broth not supplemented with any chemical was used as a control. Samples containing control and lactic acid were autoclaved at 121°C for 15 min. Copper solution was filter sterilized through 0.2-*μ*m Nalgene filtration product (Nalge Nunc International, Rochester, NY, USA) and added to the BHI broth. 

### 2.4. Measuring Bacterial Growth

Bacterial growth was monitored by measuring the turbidity at 8 h incubation period using a Spectronic 21 Melton Roy spectrophotometer (Thermo Electron Scientific Co., Madission, Wis, USA) at the wavelength of 610 nm. At the end of the incubation (8 h), one ml of each treated bacterial suspension was serially (1 : 9) diluted in sterile 0.1% (w/v) peptone solution and 100 *μ*L of appropriated dilution was surface plated on duplicate BHI agar plates. The plates were incubated at 37°C for 24 h, and bacterial colonies were counted. 

### 2.5. Determination of pH

The pH values of samples containing copper (20 and 40 ppm, w/v), lactic acid (0.2% v/v), and their combination were measured. The pH meter (Accument Excel XL15, Thermo Fisher Scientific, Pittsburgh, Pa, USA) was first calibrated with standard buffers of pH 4.0 and 10.0, and the values were recorded for each sample. 

### 2.6. Produce Preparation

Romaine lettuce (*Lactuca sativa L. var. longifolia*) and Roma tomatoes (*Lycopersicum esculentum*) were purchased at a local grocery store (Greensboro, NC, USA) and stored at 4°C the day before testing. Fresh whole tomatoes of similar sizes weighing between 55–65 g each, and without external defects on the skin, were selected and washed with tap water to remove external dirt. Two or three outer leaves of lettuce plant were discarded and a few undamaged inner leaves were selected before the washing procedure. The internal unwilted portions of leaves were cut into pieces of 4 cm by 4 cm for inoculation. Tomatoes and pieces of lettuce leaves were then dried at room temperature for 1 h in the biosafety cabinet (Labanco Corporation Kansas City, MO, USA) to remove moisture from the surface. 

### 2.7. Produce Inoculation

Whole tomatoes and pieces (4 × 4 cm) of lettuce leaves were then submerged into 400 mL of inoculum mixture (*∼*9 log CFU/mL), prepared as described earlier, and gently agitated by hand with a stainless steel spoon for 2 min at room temperature (22°C) to ensure uniform inoculation. Each produce type was inoculated separately in 400 mL of inoculum in 1000-mL beakers. To increase the number of cells attached, the samples were kept at 4°C for 12 h in sterile bags. All produce was then air dried in a laminar flow hood, on a sterile petri dish, for 1 h at room temperature, to facilitate bacterial adhesion before treatment. 

### 2.8. Preparation of Treatment Solutions

Fresh solutions of copper and lactic acid were prepared the same day as each experiment. Copper stock solution of 1000 ppm was prepared (3.93 g CuSO_4_·5H_2_O per liter) and filter sterilized through a 0.2-*μ*m Nalgene filteration product. The appropriate volume was then added to the treatment solution to give final concentrations of 20 and 40 ppm copper. A solution containing copper (20 and 40 ppm), lactic acid (0.2%), and their combination (20 ppm Cu + 0.2% La and 40 ppm Cu + 0.2% La) with and without 0.1% v/v Tween 80 (Fisher Scientific, Fair Lawn, NJ, USA) and Tween 80 only were prepared in deionized water. All solutions without copper were sterilized at 121°C for 15 min. 

### 2.9. Rinse Treatment

The washing method described by Lang et al. [26] and Velázquez et al. [2] was used in this study with slight modification. After air drying, 12 pieces of lettuce leaves and 12 whole tomatoes were individually placed inside sterile plastic bags and then rinsed with 10 mL of each treatment. Treatments included 20 ppm copper, 40 ppm copper, 0.2% lactic acid, 0.1% Tween 80, and combination of copper and lactic acid with and without Tween 80. The bags were gently shaken, and each tomato was hand rubbed for 1 min to facilitate wetting by the treatment solution. Bags with lettuce leaves were also hand agitated for one min and shaken at 100 rpm (Orbit shaker, Lab-Line instruments, Inc. Melrose Park, Ill, USA) for 3 min. Two bags containing inoculated samples of lettuce and tomato without any treatment were considered as control samples. After washing, each sample (treated and control) was then transferred to a new sterile bag and subjected to a 10 mL of 0.1% peptone solution and agitated for 1 min to release bacteria [2, 26]. 

### 2.10. Microbiological Analysis

One ml of wash suspension from each bag was serially diluted with 9 mL of sterile 0.1% peptone solution, and 100 *μ*L of appropriate dilution was plated on duplicate BHI agar. The plates were incubated at 37°C for 24 h, and bacterial colonies were counted. Typical creamy whitish colonies characteristic of *E. coli* O157:H7 were only counted. Bacterial populations were reported as log CFU/lettuce leaf or log CFU/tomato [2]. 

### 2.11. Scanning Electron Microscopy (SEM)

The morphological changes of bacterial cells treated with or without copper/lactic acid were investigated using SEM. Bacterial cells were washed with phosphate-buffered saline (PBS; 50 mM, pH 7.4), fixed with 5% glutaraldehyde at 4°C for 2 h, then washed in the buffered solution to remove the glutaraldehyde. Next, samples were dehydrated with a series of ethanol solutions (50%, 70%, 80%, 90%, and 100%) with 10 min of exposure per concentration. The dehydrated samples were dried immediately, and mounted on SEM stubs and sputter coated (Pello model 3, Sputter coater 91000, Ted Pella Inc., NY, USA) with a thin layer of gold. The coated samples were observed under SEM (Hitachi S4800, Hitachi Ltd, Tokyo, Japan) at a voltage of 15 kV. 

### 2.12. Statistical Analysis

Different treatments were statistically analyzed for their effects on *E. coli* O157:H7 by a factorial analysis of variance of duplicate samples. Each experiment was replicated three times to determine the effect of copper alone and in combination with lactic acid on the survival and growth of the bacteria. Data were analyzed using the general linear models procedure of the Statistical Analysis Software (SAS Institute, Cary, NC, USA) with the use of Duncan's multiple range to determine significant differences among the treatments (*P* < 0.05). 

## 3. Results

### 3.1. Study in Laboratory Medium

Table 1 shows the effects of copper alone or in combination with lactic acid on the growth of *E. coli *O157:H7 strains in BHI broth samples. When *E. coli*  O157:H7 strains were grown in BHI broth alone (control samples), the bacterial strains continued to grow from initial turbidity (*∼*0.03, O.D. 610 nm) and reached maximum absorbance of 0.78–0.84 within 8 h of incubation at 37°C. When copper was added at 5 ppm and 10 ppm, a slight inhibition was observed as measured by turbidity (0.76–0.82). Further addition of 20 ppm and 40 ppm copper also showed slight growth inhibition (O.D. 0.65–0.78). This indicated that the presence of copper alone had a slight effect on the growth inhibition of *E. coli *O157:H7. When lactic acid was added to BHI broth at 0.1% or 0.2% lactic acid, slight growth inhibition was observed. The addition of 5 ppm or 10 ppm copper in combination with 0.1% and 0.2% lactic acid did not show additional growth inhibition. However, when copper was added (20 ppm or 40 ppm) in combination with 0.1% and 0.2% lactic acid, a significant growth inhibition was observed. Addition of copper at 20 and 40 ppm with 0.2% lactic acid caused higher growth inhibition of the tested strains (*P* < 0.05) when compared with control or individually treated samples. The pH values of the BHI broth samples treated with 20 ppm and 40 ppm copper in combination with 0.2% lactic acid were 5.69 and 5.48, respectively, indicating that acid is not the main inhibiting factor in this study. 

Table 2 shows the populations of *E. coli* O157:H7 strains (log CFU/mL) grown in BHI broth containing different concentrations of copper and lactic acid, alone or in combination, during incubation at 37°C for 8 h. In the control samples, the numbers of *E. coli* strains increased from an initial population of 3 log CFU/mL and reached an average of 9.93 log CFU/mL. With the addition of copper at different concentrations, the growth of *E. coli* O157:H7 was not significantly inhibited (*P* > 0.05). Therefore, copper alone did not significantly inhibit the tested strains. Growth in BHI broth containing 0.1% lactic acid was not significantly affected compared to growth in the control or BHI containing only copper. When *E. coli* O157:H7 was grown in BHI broth containing four different concentrations of copper and in combination with 0.1% lactic acid, the growth of bacterial populations were slightly inhibited compared to the control and copper alone samples. Maximum of 1.09 log reduction was achieved when 40 ppm copper was supplemented with 0.1% lactic acid when compared to the lactic acid only sample. When *E. coli *O157:H7 was grown in BHI broth containing 0.2% lactic acid alone, or in combination with 5 and 10 ppm copper, reductions in the bacterial population of 2.91, 3.59 and 3.73 log CFU/mL compared to the control were not significantly different from each other. However, when a combination of 20 ppm copper with 0.2% lactic acid was used, 4.37 log CFU/mL reduction in bacterial population was achieved compared to the control sample. Furthermore, with the addition of 40 ppm copper to 0.2% lactic acid, the bacterial population was reduced by 5.03 log CFU/mL compared to the control (9.93 log CFU/mL). Therefore, 20 and 40 ppm of copper in combination with 0.2% of lactic acid is the most effective treatment to inhibit the growth of *E. coli* O157:H7 in BHI media. 

### 3.2. Effect on Produce Surface

**Figure 1:**
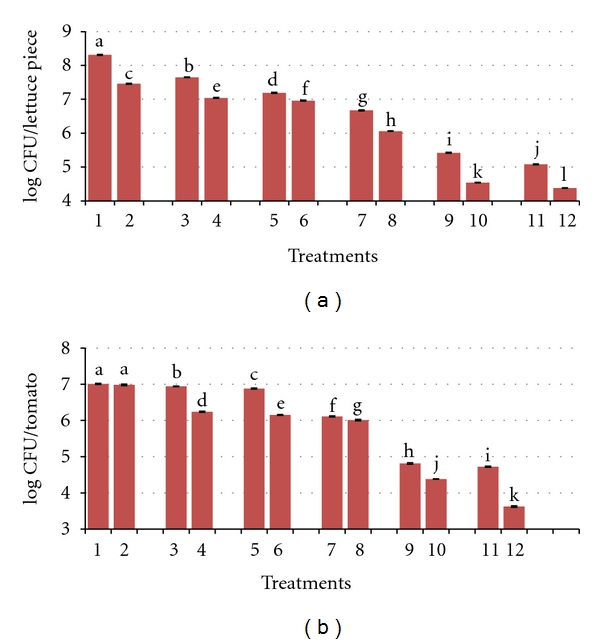
Populations of *E. coli* O157:H7 on the surface of (a) lettuce and (b) tomato samples treated with copper (Cu) and lactic acid (La) solution with and without Tween 80 (T80) as surfactant. The error bars represent the standard deviations for replicate samples. Means with different letters over the error bars are significantly different (*P* < 0.05). (1) Control, (2) Tween 80, (3) Cu 20 ppm, (4) Cu 20 ppm + T80, (5) Cu 40 ppm, (6) Cu 40 ppm + T80, (7) La 0.2%, (8) La 0.2% + T80, (9) La 0.2% + Cu 20 ppm, (10) La 0.2% + Cu 20 ppm + T80, (11) La 0.2% + Cu 40 ppm, and (12) La 0.2% + Cu 40 ppm + T80.

Figures 1(a) and 1(b) show the populations of *E. coli* O157:H7 on the surface of lettuce and tomatoes samples treated with copper alone or in combination with lactic acid solution. The initial inoculum concentration of mixed strains of *E. coli* O157:H7 was approximately 9 log CFU/mL. The microbial population in untreated samples (control) was 8.31 CFU/lettuce piece and 7.01 CFU/tomato. Rinsing lettuce pieces and whole tomatoes with copper 20 ppm solution alone resulted in 0.66 log and 0.07 log reductions of *E. coli* O157:H7, respectively, while treatment with 40 ppm copper resulted in 1.12 log and 0.13 log reductions on lettuce and tomato surfaces, respectively. Samples when rinsed with 0.2% lactic acid slightly reduced the number of microbial populations on both lettuce and tomato surfaces than the control samples. When food samples were rinsed with a combination of 20 ppm copper and 0.2% lactic acid, 2.89 log and 2.2 log reductions were achieved per lettuce piece and tomato, respectively. However, with the treatment of 40 ppm copper and 0.2% lactic acid, 3.23 and 2.29 log reductions of *E. coli* O157:H7 were achieved from lettuce and tomato surfaces, respectively. Our results demonstrated that the addition of treatment with 40 ppm copper in combination with 0.2% lactic acid significantly (*P* < 0.05) reduces the bacterial population on the surface of contaminated produce.

We also observed the impact of copper in combination with lactic acid and Tween 80 as surfactant. There was less than 1 log reduction when the lettuce surface was treated with 0.1% Tween 80 alone, and there was no significant difference between the treated tomato surface and untreated sample. Lettuce treated with copper 20 and 40 ppm with Tween 80, produced more than 1 log reduction. However, less than 1 log reduction was achieved from tomato surfaces than from lettuce undergoing the same treatment. With the addition of lactic acid (0.2%) to Tween 80 (0.1%), reductions of 2.25 log CFU/lettuce piece and 1 log CFU/tomato were observed. Similarly, less than 1 log bacterial populations were achieved from both lettuce and tomato samples rinsed with the individual treatments of copper and lactic acid in the presence of Tween 80 as compared with individual treatments without Tween 80. Lettuce and tomato treated with 20 ppm copper and 0.2% lactic acid, plus 0.1% Tween 80, reduced bacterial populations by 3.77 log and 2.63 log, respectively. When samples were treated with a 40 ppm copper and both 0.2% lactic acid and Tween 80, higher microbial reductions on both produce surfaces were achieved. The populations on lettuce and tomato surfaces were reduced by 3.93 log CFU/lettuce piece and 3.39 log CFU/tomato, respectively. 

## 4. Bacterial Morphology

**Figure 2:**
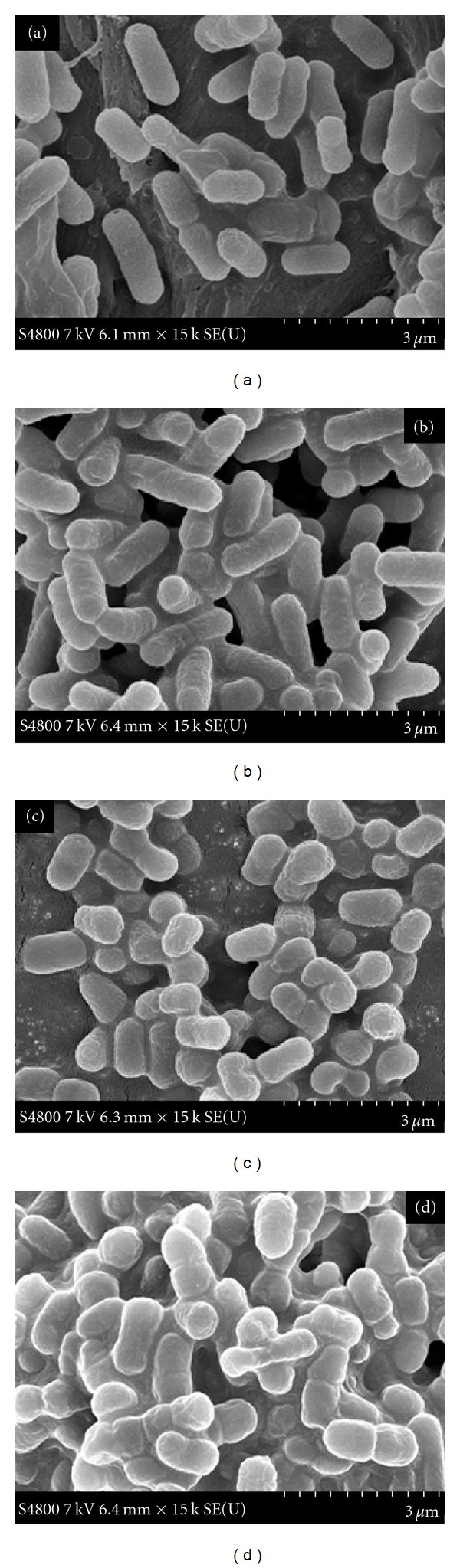
Effect of lactic acid and copper on the morphology of *E. coli* O157:H7 as demonstrated by scanning electron microscopy: (a) control, (b) lactic acid, (c) copper, and (d) lactic acid and copper.

**Figure 3:**
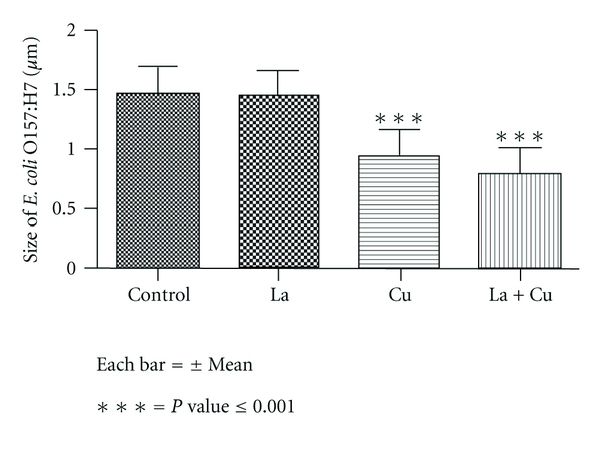
Histogram showing size (*μ*m) of *E. coli* O157:H7 after treated with copper and lactic acid.

Figure 2 shows the impact of copper alone or in combination with lactic acid on the morphology of *E. coli* O157:H7 using SEM. The bacterial cells (*E. coli* O157:H7) in the control samples (without treatment) (Figure 2(a)) showed a normal rod shape with smooth surface. Morphological (shape, size, and appearance) changes in bacterial cells were not different when treated with lactic acid (Figure 2b). On the contrary, cells grown in copper alone or in combination with lactic acid (Figures 2(c) and 2(d)) were more abnormal in shape and size indicating a disruption of the membrane integrity. In fact, the morphology of these cells (Figures 2(c) and 2(d)) revealed wrinkled abnormalities with numerous small clefts distributed around the cell surfaces.  Figure 3 shows the size distribution histogram of treated and untreated cells. The average size of untreated cells (control) and cells treated with lactic acid were not found to be different. On the other hand, cells treated with copper alone or in combination with lactic acid showed a significant difference (*P* < 0.001) in their sizes. The average cell size reduced from 1.44 *μ*m (control) to 0.81 *μ*m when treated with copper in combination with lactic acid.

## 5. Discussion

Previous studies showed that *E. coli* O157:H7 was implicated in several outbreaks involving acidic fruits and fruit juices at pH value of 3.4 [27, 28]. *E. coli* O157:H7 has also survived well in beef slurries with lactic acid [29], raising concern about the tolerance of *E. coli* O157:H7 to low pH. Therefore, it is very important to control the growth of these pathogens. Our work demonstrated that treatment with copper in combination with lactic acid showed a synergistic effect against *E. coli* O157:H7. Similar results were also obtained with *E. coli* O157:H7 in BHI broth and carrot juice in the presence of copper (50 ppm) and lactic acid (0.2%), indicating a significant inhibitory effect of copper and lactic acid [12]. Based on the results obtained from these two studies in laboratory medium, 40 ppm copper and 0.2% lactic acid could be used as a potential sanitizer to decontaminate produce surfaces. It has been demonstrated previously that the efficacy of copper ion in killing microorganisms is greatly enhanced by the use of lactic acid. Hence, the use of copper in combination with lactic acid might be an effective method to inhibit pathogenic bacteria from the surfaces of produce. Therefore, 20 ppm or 40 ppm copper in combination with 0.2% lactic acid could be used to control the growth of *E. coli* O157:H7.

Results showed that the treatment with Tween 80 alone or in combination with copper or lactic acid alone did not effectively reduce the bacterial reductions from the produce surfaces. However, a combination of copper and lactic acid plus Tween 80 effectively reduced populations of *E. coli* O157:H7 on the produce samples. Even though washing with chlorinated water is the most widely used method to inactivate pathogenic bacteria on the surfaces of fresh produce, the efficacy of chlorinated water is minimal and can only achieve approximately 2 to 3 log reductions of native microflora [30, 31]. Zhang and Farber [32] showed 1.7 and 1.2 log cfu/g reduction of *L*. *monocytogenes *on lettuce and cabbage treated with 200 ppm of chlorine, respectively. Similarly, conventional fresh cut cilantro production uses chlorinated water at 100 mg/L to decontaminate cilantro leaves [33]. Wei et al. [34] reported that chlorinated water up to 200 ppm is applied to reduce microbial contamination in produce processing lines. Despite chlorine's antimicrobial activity and low cost, the ever-increasing adverse effects of chlorine by-products have raised concerns in the food industry [33]. Furthermore, chlorine can lead to the formation of potentially carcinogenic and teratogenic trihalomethanes and haloacetic acids [35]. The results of our study indicate that 40 ppm copper with 0.2% lactic acid in the presence of Tween 80 was significantly more effective (*P* < 0.05) in removing pathogenic bacteria from produce surfaces and could be a safer alternative. Slightly larger populations of *E. coli* O157:H7 were recovered from lettuce samples than tomatoes, which may be due to the differences in their surface structure. When Tween 80 was added to the treatment solution, a higher bacterial reduction was achieved on the surface of lettuce and tomatoes. Tween 80 is an ionic surfactant approved by the US Food and Drug Administration (FDA) and also generally recognized as a safe (GRAS) product. Addition of Tween 80 might have enhanced the lethality of copper and lactic acid solution by increasing the surface contact of the solution with the microbes, thereby maximizing the release of pathogens from inoculated lettuce and tomato surfaces. Our results in Tables 1 and 2 show 5 log reduction in bacterial population that could be achieved with a combination of 40 ppm copper and 0.2% lactic acid. This indicates that the combination of 40 ppm copper and 0.2% lactic acid produced a significant reduction in *E. coli* O157:H7 in laboratory media to achieve a 5 log reduction recommended by Food and Drug Administration [36]. Therefore, results of this study suggest that further research should explore the antimicrobial effect of copper in combination with lactic acid and other natural ingredients to achieve a minimum 5 log reduction of bacterial population on the surface of fresh produce as well. 

Many studies in the past have reported that various antimicrobial agents have altered the morphology of bacterial cells. Transmission electron microscopy (TEM) has shown that *E. coli* O157:H7 cells exposed to lactoferrin and lysozyme become enlarged and hypodense, indicating bacterial killing through osmotic damage [37]. In another study observed by TEM, bacteria treated with essential oils (Eos) showed formation of blebs, coagulation of cytoplasmic constituents, damaged cell structure, and were devoid of cytoplasmic material [38]. Similarly, SEM study of bacterial cells showed damage to the outer membrane and morphological changes of the cells when treated with eicosapentaenoic acid (EPA) and potassium salt of conjugated linoleic acid (CLA-K) [39, 40]. Bacterial cells exposed to carvacrol and thymol showed the disintegration of outer membrane of *E.coli* O157:H7 and *Salmonella *Typhimurium. Disruption of the cell wall with roughness and lack of cytoplasm have been reported in *Listeria monocytogenes* when treated with thyme Eos [41]. Turgis et al. [42] showed physiological and morphological changes observed by electronic microscopy in *E. coli* O157:H7 and *S. typhi* caused by mustard EO, suggesting permeability of bacterial cells. The SEM, TEM, and Raman spectroscopy images indicate that zinc oxide nanoparticles may damage the bacterial cell membrane resulting in loss of intracellular components and eventually the death of cells [43]. In our current study, the morphological changes in *E. coli* O157:H7 caused by the action of copper and lactic acid are somehow similar to the observations made by previous studies [39–42]. The study of bacterial populations in laboratory medium did not show any effect of copper alone against *E. coli* O157:H7. However, the SEM study showed that copper alone can alter the cell morphology, and this could be considered as the initial stage of inhibition that may lead to the ultimate death of cells. Significant reduction (*P* ≤ 0.001) of cell size with combination treatment was probably due to the acidic permeability effect on the cell membrane. This effect on permeability could have further enhanced the antimicrobial efficacy of copper against bacterial cells. 

The molecular mechanism by which copper and lactic acid affect antimicrobial activity is not well understood. Various authors have hypothesized that organic acids may damage the outer or cytoplasm cell membrane or denature proteins and DNA [44, 45]. Alakomi et al. [25] reported that the increase of permeability of bacteria membranes may potentiate the effect of other antimicrobial agents as shown with lactic acid and sodium laurylsulphate. Microorganisms including bacteria require copper at low concentrations as essential micronutrients, but, at high concentrations, copper can cause inhibition of cell growth or even death of the cells [11]. Helander and Mattila-Sandholm [46] found that an increase in cell wall and plasma membrane permeability can sensitize bacteria to an antimicrobial agent that alone is unable to penetrate into deeper bacterial targets. Therefore, one possible explanation of cell death could be that it is due to the changes of membrane permeability caused by the use of acid. It is possible that lactic acid may have permeabilized the cell membrane, allowing copper ions into the bacterial cells and thus producing a toxic effect. However, our SEM study does not show any type of inner cell membrane damage or denaturation of protein. We only observed differences in cell's shape and size. This finding suggests that further research inside the microorganism cell could provide additional knowledge about the extent of damage and the mode of action of copper and lactic acid against *E. coli* O157:H7. Transmission electron microscopy (TEM) can be used to observe bacterial ultrastructural alterations in detail. Furthermore, we believe that this study represents the first published work to demonstrate the antimicrobial activity of a copper in combination with lactic acid solution as a sanitizing agent. Copper in combination with lactic acid may produce a synergy that reduces the number of pathogenic microorganisms, including *E. coli *O157:H7, on the surfaces of lettuce and tomatoes. This solution could be a potential decontaminant for fresh produce. In conclusion, our study in laboratory medium and SEM has shown that copper in combination with lactic acid possesses antimicrobial activity against Gram-negative bacteria such as *E. coli* O157:H7. The growth inhibition confirmed by measuring bacterial growth and morphological changes in cell size observed from SEM images indicates the potential use of copper and lactic acid against *E. coli* O157:H7. Therefore, copper in combination with lactic acid could be potentially considered to be an effective antibacterial agent for enhancing agricultural and food safety. 

## Figures and Tables

**Table 1:** Growth of *E. coli* O157:H7 strains in BHI broth (O.D.) after incubation for 8 h at 37°C in the presence of (Cu) and lactic acid (La).

*E. coli* O157:H7 strains				

Treatments	H1730	43895+	43895−	86.24

O.D. 610 nm				
Control	0.84^a^ ± 0.064	0.81^a^ ± 0.014	0.78^a^ ± 0.035	0.83^a^ ± 0.021
Cu 5 ppm	0.81^ab^ ± 0.035	0.78^ab^ ± 0.007	0.77^ab^ ± 0.021	0.82^ab^ ± 0.042
Cu 10 ppm	0.80^ab^ ± 0.014	0.76^bc^ ± 0.014	0.77^ab^ ± 0.007	0.79^bc^ ± 0.028
Cu 20 ppm	0.78^b^ ± 0.028	0.74^bc^ ± 0.014	0.74^ac^ ± 0.014	0.75^cd^ ± 0.014
Cu 40 ppm	0.76^bc^ ± 0.014	0.71^de^ ± 0.042	0.65^d^ ± 0.007	0.69^ef^ ± 0.007
La 0.1%	0.71^cd^ ± 0.028	0.69^e^ ± 0.021	0.71^c^ ± 0.049	0.72^de^ ± 0.014
La 0.1% + Cu 5 ppm	0.70^cd^ ± 0.014	0.67^ef^ ± 0.021	0.71^c^ ± 0.021	0.69^ef^ ± 0.049
La 0.1% + Cu 10 ppm	0.66^ed^ ± 0.007	0.65^f^ ± 0.014	0.71^c^ ± 0.007	0.69^ef^ ± 0.000
La 0.1% + Cu 20 ppm	0.63^ef^ ± 0.000	0.61^g^ ± 0.021	0.65^d^ ± 0.014	0.66^f^ ± 0.028
La 0.1% + Cu 40 ppm	0.61^efg^ ± 0.028	0.55^h^ ± 0.021	0.51^ef^ ± 0.021	0.58^g^ ± 0.007
La 0.2%	0.58^fg^ ± 0.021	0.50^i^ ± 0.007	0.54^e^ ± 0.007	0.60^g^ ± 0.000
La 0.2% + Cu 5 ppm	0.55^g^ ± 0.071	0.49^i^ ± 0.014	0.48^f^ ± 0.021	0.52^h^ ± 0.000
La 0.2% + Cu 10 ppm	0.48^h^ ± 0.014	0.44^j^ ± 0.007	0.38^g^ ± 0.007	0.50^h^ ± 0.007
La 0.2% + Cu 20 ppm	0.28^i^ ± 0.028	0.35^k^ ± 0.014	0.28^h^ ± 0.007	0.36^i^ ± 0.028
La 0.2% + Cu 40 ppm	0.11^j^ ± 0.028	0.26^l^ ± 0.021	0.17^a^ ± 0.007	0.26^j^ ± 0.021

Means (± standard deviation) within the same column not followed by the same letters are significantly different (*P* < 0.05).

Initial bacterial population was approximately 3.00 log CFU/mL.

**Table 2:** Populations of *E. coli* O157:H7 strains grown in BHI broth (Log CFU/mL) containing different concentrations of copper (Cu) and lactic acid (La) after incubation for 8 h at 37°C.

*E. coli* O157:H7 strains					

Treatments	H1730	43895+	43895−	86.24	Average

Log CFU/mL					
Control	10.42	10.40	9.37	9.55	9.93^a^ ± 0.55
Cu 5 ppm	10.28	10.35	9.34	9.45	9.85^a^ ± 0.53
Cu 10 ppm	10.25	10.12	9.29	9.42	9.77^a^ ± 0.48
Cu 20 ppm	9.70	9.20	9.08	9.25	9.30^ab^ ± 0.27
Cu 40 ppm	9.51	9.17	8.98	9.10	9.19^ab^ ± 0.22
La 0.1%	9.45	9.68	8.75	8.45	9.08^abc^ ± 0.56
La 0.1% + Cu 5 ppm	9.40	9.55	7.86	7.60	8.60^bc^ ± 1.02
La 0.1% + Cu 10 ppm	9.31	9.25	7.82	7.30	8.42^bc^ ± 1.01
La 0.1% + Cu 20 ppm	9.15	8.93	7.72	6.95	8.18^bc^ ± 1.03
La 0.1% + Cu 40 ppm	9.10	8.34	7.66	6.87	7.99^cd^ ± 0.95
La 0.2%	7.81	7.30	6.15	6.84	7.02^de^ ± 0.70
La 0.2% + Cu 5 ppm	7.49	6.90	5.95	5.02	6.34^ef^ ± 1.08
La 0.2% + Cu 10 ppm	7.11	6.82	5.90	5.00	6.20^ef^ ± 0.95
La 0.2% + Cu 20 ppm	6.03	6.02	5.55	4.66	5.56^fg^ ± 0.64
La 0.2% + Cu 40 ppm	5.46	5.51	4.56	4.10	4.90^gh^ ± 0.69

Means (± standard deviation) within the same column not followed by same letters are significantly different (*P* < 0.05).

Initial bacterial population was approximately 3.00 log CFU/mL.
